# N-phenethylacetamide, diaminopimelic acid, and Gly-Val as high-performance serum biomarkers for diagnosing untreated Graves’ disease: an LC-MS-based metabolomics study

**DOI:** 10.3389/fendo.2025.1707049

**Published:** 2025-11-11

**Authors:** Lihua Fang, Qing Ning, Chaowen Wu, Dan Liu, Jie Ning

**Affiliations:** 1 Department of Endocrinology, Shenzhen Longhua District Central Hospital, Shenzhen, Guangdong, China; 2 The Second School of Clinical Medicine, Southern Medical University, Guangzhou, Guangdong, China

**Keywords:** Graves’ disease, serum, LC-MS, metabolomics, biomarker, N-phenethylacetamide, diaminopimelic acid, Gly-Val

## Abstract

**Introduction:**

Graves' disease (GD), a common autoimmune thyroid disorder, is typified by hyperthyroidism and pervasive metabolic perturbations. Metabolomics, a burgeoning field instrumental in biomarker identification and elucidating systemic biological mechanisms, has recently shed light on the intricate pathophysiology of GD. The present study endeavors to delineate the metabolic aberrations in untreated GD patients from Shenzhen, China, leveraging LC-MS-based serum metabolomics.

**Methods:**

A cohort comprising 30 newly diagnosed, untreated GD patients and 32 healthy controls was assembled. Serum metabolite profiling was conducted via LC-MS, with subsequent identification and quantification of metabolites. Multivariate statistical analyses, encompassing principal component analysis (PCA) and partial least squares discriminant analysis (PLS-DA), were employed to discern significant metabolic discrepancies. Pathway enrichment analysis and receiver operating characteristic (ROC) curve analysis were utilized to assess the diagnostic efficacy of the identified metabolites.

**Results:**

A total of 334 significantly dysregulated metabolites were uncovered, with a pronounced involvement of lipid and organic acid metabolic pathways. Notably, N-phenethylacetamide (AUC = 0.94), diaminopimelic acid (AUC = 0.93), and the dipeptide Gly-Val (AUC = 0.91) exhibited substantial diagnostic potential. Pathway enrichment analysis unveiled significant alterations in linoleic acid, alpha-linolenic acid, and arachidonic acid metabolism, underscoring the pivotal role of inflammatory lipid pathways and amino acid metabolism in GD.

**Discussion:**

This study offers a granular metabolic profile of untreated Graves' disease, unmasking profound dysregulation within lipid and organic acid metabolism. The identified metabolites, particularly N-phenethylacetamide, diaminopimelic acid, and Gly-Val, emerge as promising high-performance serum biomarkers for GD diagnosis. These findings not only augment our comprehension of the metabolic reprogramming inherent to GD but also proffer potential targets for subsequent therapeutic endeavors. Subsequent investigations are imperative to elucidate the mechanistic roles of these metabolites in GD pathogenesis and their viability as clinical biomarkers.

## 1 Introduction

Graves’ disease (GD) is a common autoimmune disorder characterized by hyperthyroidism resulting from thyroid-stimulating hormone receptor (TSHR)-activating autoantibodies (1). It predominantly affects women and can involve extrathyroidal manifestations such as orbitopathy, driven by the synergistic action of TSHR and insulin-like growth factor 1 receptor (IGF1R) autoantibodies (2). Metabolomics is a powerful tool for biomarker discovery that can also reveal systems-level biology and detect subtle alterations in pathways, providing mechanistic insights into the disease. In recent years, advancements in metabolomics (3) have offered novel perspectives for a deeper understanding of the pathophysiology of GD, especially in the realm of serum proteins and metabolites (4). By employing proteomics and metabolomics approaches, researchers have been able to uncover alterations in proteins and metabolites within the serum of GD patients. These alterations are potentially intertwined with the disease’s pathogenesis, diagnosis, and treatment. One study utilized Mendelian randomization (MR) in conjunction with genome-wide association study (GWAS) data to scrutinize the impact of 486 serum metabolites on GD (5). It identified 19 metabolites significantly associated with GD risk. Notably, three metabolites, kynurenine, glycerol 2-phosphate, and 4-androsten-3beta,17beta-diol disulfate 2, exhibited significant heritability and lacked shared genetic correlations with GD (6). This finding underscores the potential causal significance of these metabolites in the disease. Another investigation employing untargeted metabolomics to analyze serum samples from children with GD uncovered 48 differential metabolites between the GD and control groups, encompassing amino acids, dipeptides, lipids, and purines (7). These metabolites are implicated in pathways such as aminoacyl-tRNA biosynthesis, metabolism of various amino acids, purine metabolism, and pyrimidine metabolism.

Serum metabolomic profiling of patients with GD has unveiled a distinct metabolic signature characterized by pervasive dysregulation across multiple biochemical pathways. Integrative analyses demonstrate consistent perturbations in arginine and proline metabolism, aminoacyl-tRNA biosynthesis, alanine–aspartate–glutamate axis, and bile acid homeostasis (8). Notably, these metabolic aberrations exhibit clinically significant correlations with disease manifestations, including the degree of hyperthyroidism, autoantibody titers, and overall disease severity (9). The mechanistic implications of these findings extend beyond mere association, suggesting active involvement of metabolic reprogramming in GD pathogenesis (10). Furthermore, the dynamic nature of these metabolic profiles in response to therapeutic interventions highlights their potential utility as sophisticated biomarkers for treatment monitoring and personalized therapeutic strategies (11).

Building on these advancements, the present study introduces certain improvements and, for the first time, investigates patients with newly diagnosed Graves’ disease in the Shenzhen area. By conducting a comparative analysis of serum metabolite differences, this study aims to enhance accuracy and sensitivity. It is anticipated that this research will offer more robust support for the diagnosis, treatment, and prevention of GD.

## 2 Materials and methods

### 2.1 Recruitment and study protocol

For this investigation, we enrolled 30 consecutive individuals with newly diagnosed (**Table 1**) untreated Graves’ disease (GD) and 32 healthy controls (HCs), all within the age range of 18–67 years. GD was diagnosed in accordance with the 2016 American Thyroid Association (ATA) criteria, which include suppressed TSH, elevated free T4 levels, and positive TSI, or diffuse uptake on 99mTc-pertechnetate scintigraphy. The final diagnosis was confirmed by an experienced endocrinologist, integrating all available clinical, biochemical, serological, and imaging data. This comprehensive approach ensured that all enrolled patients had a definitive diagnosis of GD, including those with atypical biochemical presentations that were clarified by positive autoimmunity or characteristic imaging findings. We excluded individuals who were pregnant or lactating; had a history of malignancy, cardiovascular disease, or diabetes; had used antibiotics or probiotics within the past 3 months; had gastrointestinal disorders and psychiatric conditions; or were taking selenium supplements.

**Table 1:** Baseline clinical and biochemical characteristics of the study cohort with untreated Graves’ disease (*n* = 30; M, male; F, female).

No.	Gender	Age	TSH (0.27–4.2) mIU/L	FT3 (3.1–6.8) pmol/L	FT4 (12–22) pmol/L	TRAb (<1.75) IU/L
1	M	21	0.008	6.94	31.06	0.98
2	F	44	<0.005	6.48	18.7	6
3	F	56	<0.005	13.30	40.85	4.64
4	F	32	<0.005	11.30	19.99	>40.00
5	M	25	<0.005	32.97	64.46	14.35
6	M	26	<0.005	10.06	25.13	2.89
7	F	43	<0.005	20.63	54.38	6.54
8	M	38	<0.005	15.09	43.70	16.76
9	M	32	<0.005	7.55	17.85	30.37
10	M	37	<0.005	13.2	26.33	9.13
11	M	40	<0.005	5.05	15.59	21.43
12	M	67	0.268	9.37	16.72	15.17
13	F	27	<0.004	>30.72	33.95	4.54
14	F	23	9.97	4.32	9.97	34.87
15	M	35	0.011	4.3	25.72	24.38
16	M	37	<0.005	34.93	78.77	17.78
17	M	36	<0.005	9.82	29.88	6.36
18	M	38	<0.005	7.44	24.84	3.74
19	M	58	<0.005	5.31	23.78	4.59
20	F	38	0.005	9.63	33.13	6.05
21	M	22	0.006	8.68	36.67	13.06
22	F	30	0.013	4.22	10.27	9.48
23	M	31	0.01	5.58	23.7	4.07
24	F	25	0.165	3.32	11.67	1.75
25	F	45	0.007	7.07	23.22	26.02
26	F	38	2.330	5.17	16.84	9.98
27	M	51	<0.005	36.09	>100.00	11.91
28	M	41	<0.005	15.24	30.24	11.21
29	M	34	<0.005	10.67	39.68	6.53
30	F	18	0.010	10.47	30.09	2.70

HCs were individuals undergoing routine health checkups, recruited from the hospital’s Department of Physical Examination. All HC participants were confirmed to be euthyroid based on clinical assessment and laboratory testing. Key inclusion criteria consisted of the absence of a personal or family history of thyroid disease, no current or previous use of medications known to affect thyroid function, and having thyroid function tests (TSH, FT3, FT4) with results within clinically acceptable normal limits. Specifically, all HC participants had TSH levels within the reference range (0.27–4.2 mIU/L). A small number of participants (*n* = 5) exhibited FT4 values slightly below the lower reference limit (12 pmol/L) but with concomitant normal TSH levels; this pattern is recognized in clinical practice as carrying no significant risk for hypothyroidism and was categorized as normal variation. Therefore, all HC participants were rigorously defined as having normal thyroid function. The study was approved by the local ethics committee under protocol number 2023-096-03, and all participants provided informed consent.

### 2.2 Sample collection and metabolite extraction

Fasting venous blood samples were collected from all participants. Serum was obtained by centrifugation and aliquoted and frozen immediately at −80°C until batch metabolite extraction. All samples underwent only one freeze–thaw cycle for this analysis. For metabolite extraction, 100 μL of serum was transferred into a 1.5-mL centrifuge tube and mixed with 400 μL of a solution composed of acetonitrile and methanol in a 1:1 volume ratio, containing an internal standard (L-2-chlorophenylalanine) at a concentration of 0.02 mg/mL. The samples were vortexed for 30 s to ensure thorough mixing and then subjected to low-temperature sonication at 5°C and 40 kHz for 30 min to facilitate the extraction process. To precipitate proteins, the samples were subsequently stored at −20°C for 30 min (12). Following this, the samples underwent centrifugation at 4°C and 13,000*g* for 15 min. The supernatant was carefully removed and evaporated to dryness under a stream of nitrogen gas (13). The dried samples were then reconstituted in 100 μL of a solution consisting of acetonitrile and water in a 1:1 volume ratio. This reconstituted solution was further processed by low-temperature ultrasonication at 5°C and 40 kHz for 5 min, followed by another centrifugation step at 4°C and 13,000*g* for 10 min. The final supernatant was carefully transferred to sample vials, which were then prepared for LC-MS/MS analysis (14).

### 2.3 Quality control procedures

To ensure the reliability and stability of the analytical process, a pooled quality control (QC) sample was created by combining equal volumes from all individual samples. This QC sample was subjected to the same preparation and analytical procedures as the experimental samples. Its purpose was to provide a representative benchmark for the entire sample set. The QC sample was injected at regular intervals (every 5–15 samples) throughout the analysis to continuously monitor and ensure the consistency and stability of the analytical system.

### 2.4 UPLC-MS/MS analysis

The UPLC-MS/MS analysis was performed using a Thermo UHPLC-Exploris 240 system equipped with an ACQUITY HSS T3 column (100 mm × 2.1 mm i.d., 1.8 μm; Waters, USA) at Majorbio Bio-Pharm Technology Co. Ltd. (Shanghai, China) (15). The mobile phases used were 0.1% formic acid in water:acetonitrile (95:5, v/v) (solvent A) and 0.1% formic acid in acetonitrile:isopropanol:water (47.5:47.5:5, v/v) (solvent B). The gradient elution for positive ion mode was as follows: 0–3 min, solvent B increased from 0% to 20%; 3–4.5 min, solvent B increased to 35%; 4.5–5 min, solvent B increased to 100%; 5–6.3 min, solvent B maintained at 100%; 6.3–6.4 min, solvent B decreased to 0%; and 6.4–8 min, solvent B maintained at 0%. For negative ion mode, the gradient was as follows: 0–1.5 min, solvent B increased from 0% to 5%; 1.5–2 min, solvent B increased to 10%; 2–4.5 min, solvent B increased to 30%; 4.5–5 min, solvent B increased to 100%; 5–6.3 min, solvent B maintained at 100%; 6.3–6.4 min, solvent B decreased to 0%; and 6.4–8 min, solvent B maintained at 0%. The flow rate was set at 0.40 mL/min, and the column temperature was maintained at 40°C.

### 2.5 Mass spectrometry conditions

The mass spectrometric data were collected using a Thermo UHPLC-Exploris 240 Mass Spectrometer equipped with an electrospray ionization (ESI) source, operating in both positive and negative modes. The optimal conditions were set as follows: auxiliary gas heating temperature at 350°C, capillary temperature at 320°C, sheath gas flow rate at 60 psi, auxiliary gas flow rate at 20 psi, ion-spray voltage floating (ISVF) at −3,000 V in negative mode and 3,400 V in positive mode, and normalized collision energy set to 20–40–60 eV for MS/MS. The full MS resolution was 60,000, and the MS/MS resolution was 15,000. Data acquisition was performed in data-dependent acquisition (DDA) mode, with a mass range of 70–1,050.

### 2.6 Data analysis

The UHPLC-MS raw data were processed using Progenesis QI software (Waters, Milford, USA) to convert the data into a common format. This involved baseline filtering, peak identification, integration, retention time correction, and peak alignment. The resulting data matrix, containing sample names, *m*/*z* values, retention times, and peak intensities, was exported for further analysis. Metabolite identification was performed by querying the Human Metabolome Database (HMDB; http://www.hmdb.ca/), Metlin (https://metlin.scripps.edu/), and the Majorbio Database (MJDB) from Majorbio Biotechnology Co., Ltd. (Shanghai, China) (16).

The data matrix was uploaded to the Majorbio cloud platform (https://cloud.majorbio.com) for analysis. Preprocessing steps included retaining metabolic features detected in at least 80% of samples, filling missing values with the minimum value, and normalizing each metabolite’s intensity to the sum of all intensities. Variables with a relative standard deviation (RSD) >30% in QC samples were excluded, and the remaining data were log10-transformed to create the final data matrix.

Principal component analysis (PCA) and orthogonal partial least squares discriminant analysis (OPLS-DA) were performed using the R package “ropls” (version 1.6.2). Metabolites with high variable importance in projection (VIP) >1 and *p <*0.05 were considered significantly different based on the OPLS-DA model and Student’s *t*-test. Differential metabolites were mapped to biochemical pathways using the KEGG database (http://www.genome.jp/kegg/). Enrichment analysis was conducted using the Python package “scipy.stats” to identify the most relevant biological pathways.

The diagnostic performance of individual metabolites and metabolite panels was evaluated using receiver operating characteristic (ROC) curve analysis based on a random forest classification model, rather than on univariate logistic regression. This machine learning approach assesses the importance of a variable within a complex, multiparametric context.

## 3 Results

### 3.1 Multivariate statistical analyses reveal distinct metabolomic profiles between GD and HC groups

**Figure 1:**
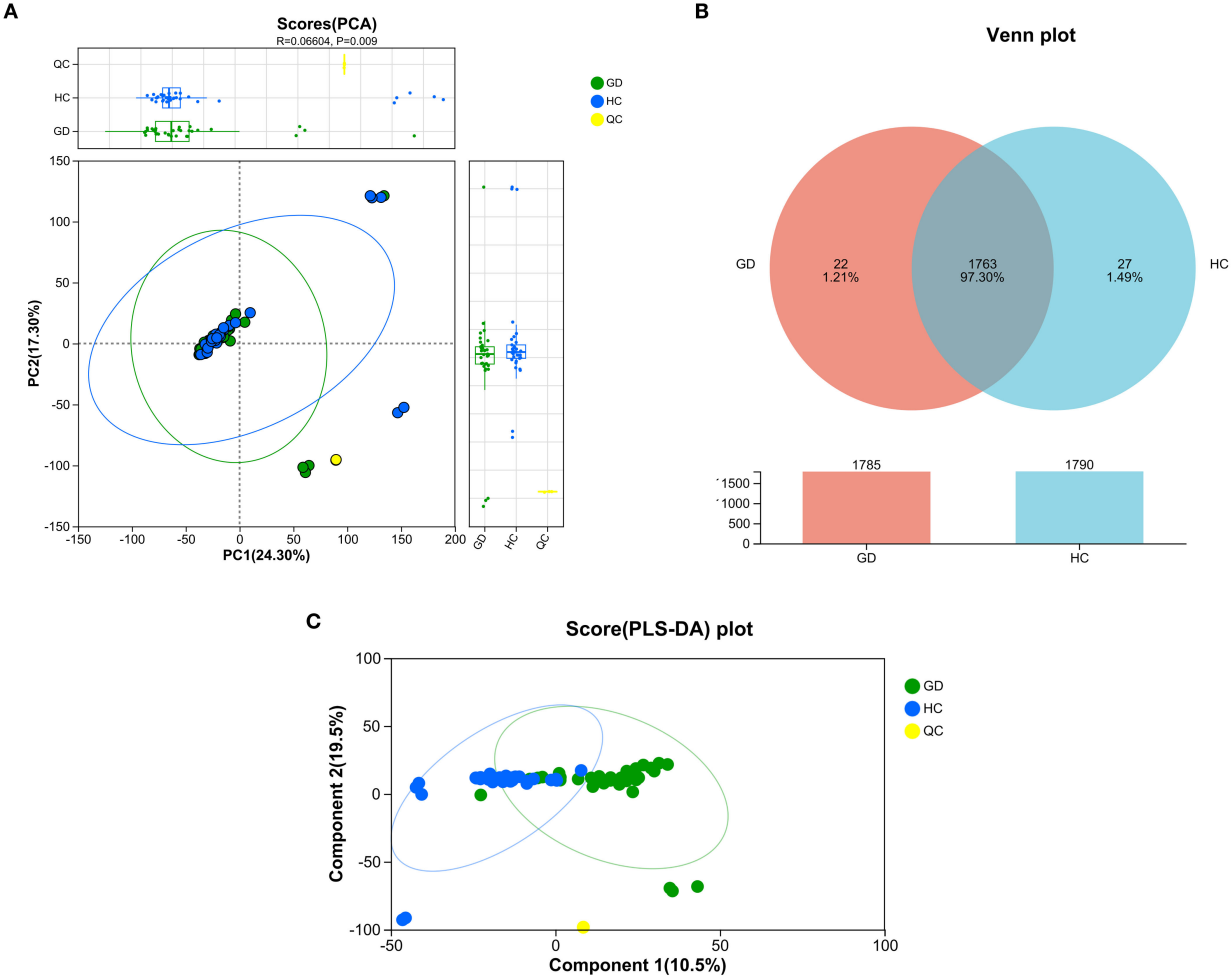
Multivariate analysis of serum metabolomic profiles from Graves’ disease patients and healthy controls. **(A)** Principal component analysis (PCA) score plot showing partial separation between GD patients, healthy controls (HCs), and quality control (QC) samples. PC1 and PC2 explain 24.30% and 17.30% of the total variance, respectively (*R*² = 0.066, *p* = 0.009). **(B)** Venn diagram illustrating the overlap and unique metabolic features between the GD and HC groups. **(C)** PLS-DA score plot demonstrating clear separation between GD and HC groups along the first two components (10.5% and 19.5% of variance, respectively).

Principal component analysis (PCA) was performed to visualize the overall distribution and grouping trends of serum metabolomic profiles among GD patients, HCs, and QC samples (**Figure 1A**). The resulting PCA score plot demonstrated a partial separation between the GD and HC groups along the principal components, with PC1 and PC2 accounting for 24.30% and 17.30% of the total variance, respectively. Although the model fit was moderate (*R*² = 0.066), the permutation test indicated statistical significance (*p* = 0.009), supporting the presence of distinct metabolic patterns between GD patients and healthy individuals. The QC samples clustered tightly in the score plot, reflecting the high reproducibility and stability of the LC-MS analytical platform throughout the experiment. These findings suggest that untreated GD is associated with significant alterations in the serum metabolome, providing a basis for further identification of potential biomarkers.

A Venn diagram was constructed to illustrate the unique and overlapping metabolic features between the GD and HC groups (**Figure 1B**). The analysis revealed 1,785 metabolites common to both groups, while 22 metabolites were uniquely expressed in the GD group and 27 were specific to the HC group. These exclusive metabolites, which account for 1.21% and 1.49% of the total detected features in GD and HC, respectively, may reflect distinct metabolic disturbances associated with Graves’ disease. The large number of shared metabolites indicates considerable metabolic consistency between groups, yet the unique features highlight potential biomarker candidates worthy of further investigation. These findings reinforce the presence of a specific metabolomic signature in GD patients, consistent with the group separation observed in PCA.

To further maximize the separation between Graves’ disease patients and healthy controls and identify the most influential metabolic variables, a supervised partial least squares-discriminant analysis (PLS-DA) was performed (**Figure 1C**). The resulting score plot demonstrated a markedly improved and clear separation between the GD and HC groups along the first two components, which together accounted for 30.0% of the total variance (component 1: 10.5%; component 2: 19.5%). This enhanced separation, compared to the unsupervised PCA model, confirms that the metabolomic profiles contain group-specific patterns that can be effectively modeled to distinguish GD from healthy controls. The distinct clustering of the QC samples again underscores the robustness and reproducibility of the analytical platform. The PLS-DA model provides a strong foundation for the subsequent identification of discriminant metabolites with high VIP scores.

### 3.2 KEGG-based annotation and enrichment highlight broad alterations in lipid and organic acid metabolic pathways

**Figure 2:**
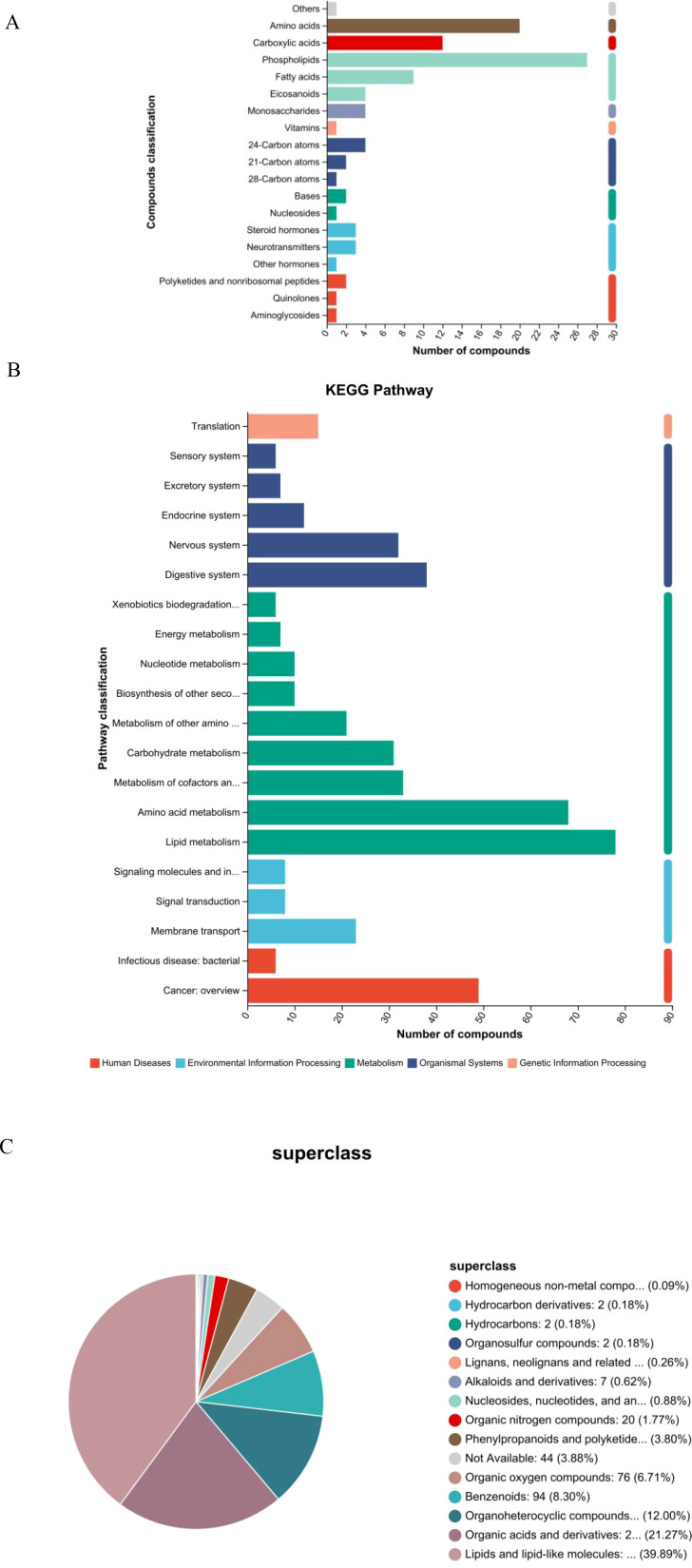
Classification and pathway analysis of differentially abundant metabolites. **(A)** Bar chart of KEGG compound classification showing the predominance of lipids and organic acids among altered metabolites in GD. **(B)** KEGG pathway enrichment analysis highlighting significantly enriched metabolic pathways, including linoleic acid and arachidonic acid metabolism. **(C)** Pie chart of HMDB superclass distribution confirming the dominance of lipids and lipid-like molecules (39.89%) and organic acids and derivatives (21.27%).

Metabolites that were significantly altered between the GD and HC groups were annotated and classified based on the KEGG compound database to understand the major classes of compounds affected (**Figure 2A**). The classification bar chart revealed that the most prominent categories of differential metabolites belonged to lipids and organic acids, underscoring a substantial disruption in these metabolic pathways in Graves’ disease. Other significantly represented classes included carbohydrates, peptides, and nucleic acids. Notably, within the lipid category, subclasses such as fatty acids, phospholipids, and steroids (including steroid hormones) were highly abundant, aligning with the known metabolic disturbances in hyperthyroid conditions. The diversity of affected compound classes, with a significant number also categorized under “others,” indicates a widespread metabolic reprogramming associated with GD, impacting energy metabolism, structural lipid composition, and signaling molecules.

To further elucidate the biological implications of the altered metabolites, pathway enrichment analysis was performed based on the KEGG database. The results, visualized in **Figure 2B**, demonstrate that the majority of the differentially abundant metabolites were significantly enriched in pathways belonging to the metabolism supercategory. Specifically, lipid metabolism and amino acid metabolism were among the most represented pathways, confirming the central role of metabolic reprogramming in GD pathogenesis. A substantial number of compounds were also mapped to pathways within organismal systems, particularly the endocrine system, which is directly relevant to the autoimmune endocrine nature of Graves’ disease. Furthermore, enrichment was observed in pathways related to human diseases and environmental information processing, including signal transduction. This comprehensive pathway analysis indicates that the metabolic disturbances in GD extend beyond core metabolism, affecting systemic regulatory and signaling networks, and provides a functional context for the identified biomarker candidates.

### 3.3 HMDB superclass analysis confirms the predominance of lipids and organic acids

To gain a broader chemical perspective on the altered metabolome, differential metabolites were classified according to the HMDB superclass system. The resulting pie chart (**Figure 2C**) demonstrates that the vast majority of these metabolites belonged to the “lipids and lipid-like molecules” superclass, accounting for 39.89% of all identified compounds. This was followed by the “organic acids and derivatives” superclass, which represented 21.27% of the total. These two dominant categories align perfectly with the findings from the KEGG-based classification, robustly confirming that dysregulation of lipid and organic acid metabolism is a core characteristic of the Graves’ disease metabolomic profile. Other notable superclasses included “organoheterocyclic compounds” (12.00%) and “benzenoids” (8.30%). A small proportion of metabolites (3.88%) were categorized as “not available,” indicating compounds that await further classification. This HMDB-based chemical taxonomy provides a high-level, chemically grounded overview that strongly supports the centrality of specific metabolic pathways in GD.

### 3.4 Volcano plot analysis and validation of key differential metabolites

**Figure 3:**
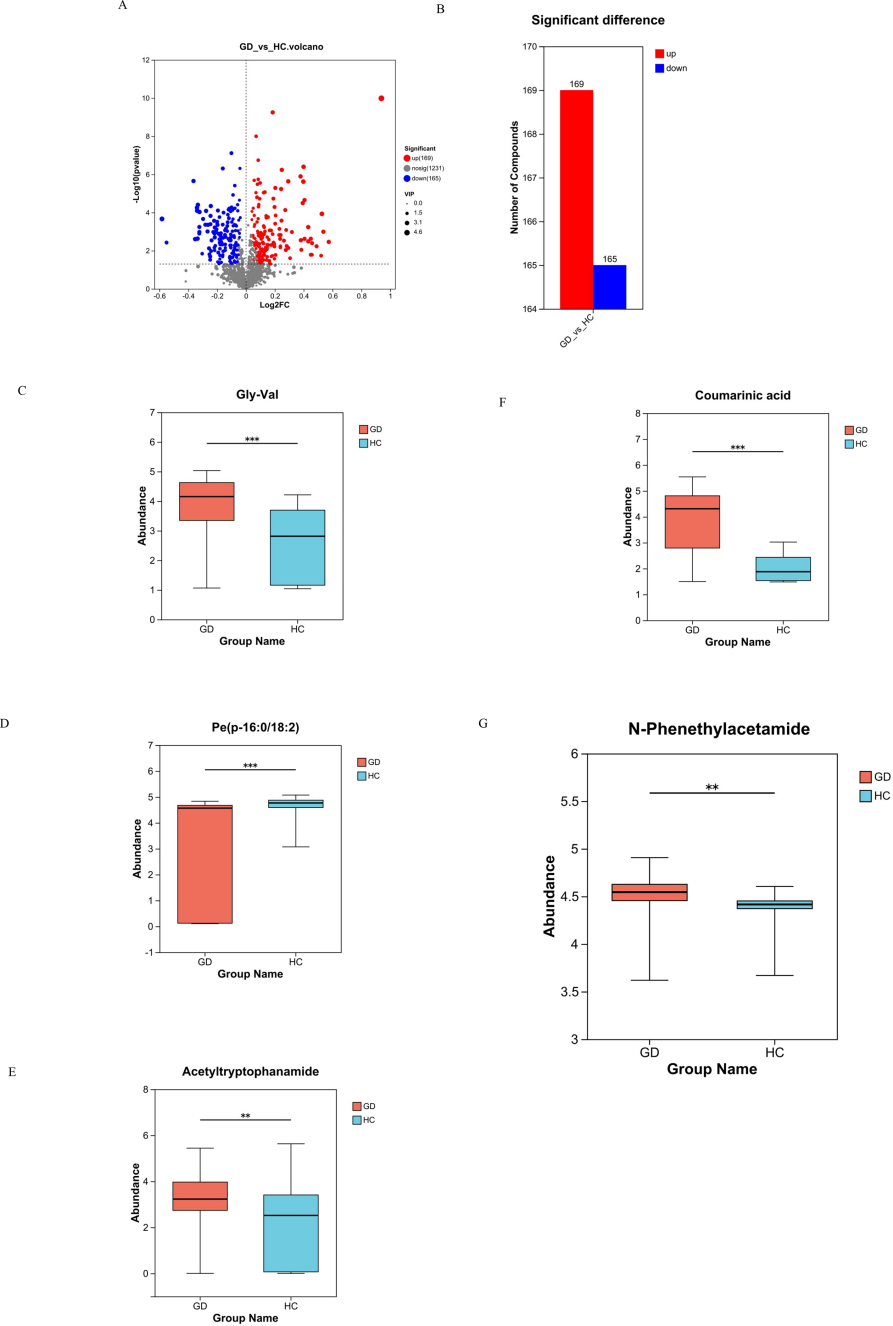
Volcano plot and box plots of key differential metabolites. **(A, B)** Volcano plot visualizing 334 significantly dysregulated metabolites (169 upregulated, 165 downregulated) in GD vs. HC. **(C–G)** Box plots comparing the abundance of selected metabolites: **(C)** Gly-Val, **(D)** PE(P-16:0/18:2), **(E)** acetyltryptophanamide, **(F)** coumaric acid, and **(G)** N-phenethylacetamide. ** 0.001 < P ≤ 0.01, *** P ≤ 0.001.

Volcano plot analysis was employed to visualize the extent and significance of metabolic changes between the GD and HC groups. The analysis identified a total of 334 significantly differentially abundant metabolites. Among these, 169 metabolites were significantly upregulated and 165 were significantly downregulated in the GD group compared to HC. This widespread dysregulation across a substantial number of compounds highlights the profound impact of GD on the serum metabolome (**Figures 3A, B**).

To confirm the identity and statistical significance of specific potential biomarkers, the abundance levels of several key differential metabolites were compared between the GD and HC groups using box plots, which revealed distinct and significant abundance patterns for each compound (**Figures 3C–G**). The dipeptide Gly-Val (glycylvaline) showed significantly altered levels in GD patients. The phospholipid species PE(P-16:0/18:2), a plasmalogen phosphatidylethanolamine, exhibited a marked difference in abundance. Acetyltryptophanamide, a tryptophan derivative, was also identified as being significantly dysregulated. Furthermore, coumaric acid, a phenolic acid, demonstrated a significant change in concentration, highlighting perturbations in related pathways. Notably, N-phenethylacetamide, which demonstrated the highest individual diagnostic accuracy (AUC = 0.940), was confirmed to be significantly upregulated in the GD group (*p* = 0.004, FDR-corrected *p* = 0.031; VIP > 1) (**Figure 3G**), robustly supporting its role as a top-tier candidate biomarker. The consistent and statistically significant alterations in the abundance of these specific compounds, which span critical chemical classes including lipids, amino acid derivatives, and phenolic acids, provide strong evidence for their involvement in the metabolic disturbances of Graves’ disease and validate them as high-priority candidate biomarkers.

### 3.5 Hierarchical clustering and VIP analysis reveal expression patterns and discriminatory metabolites

**Figure 4:**
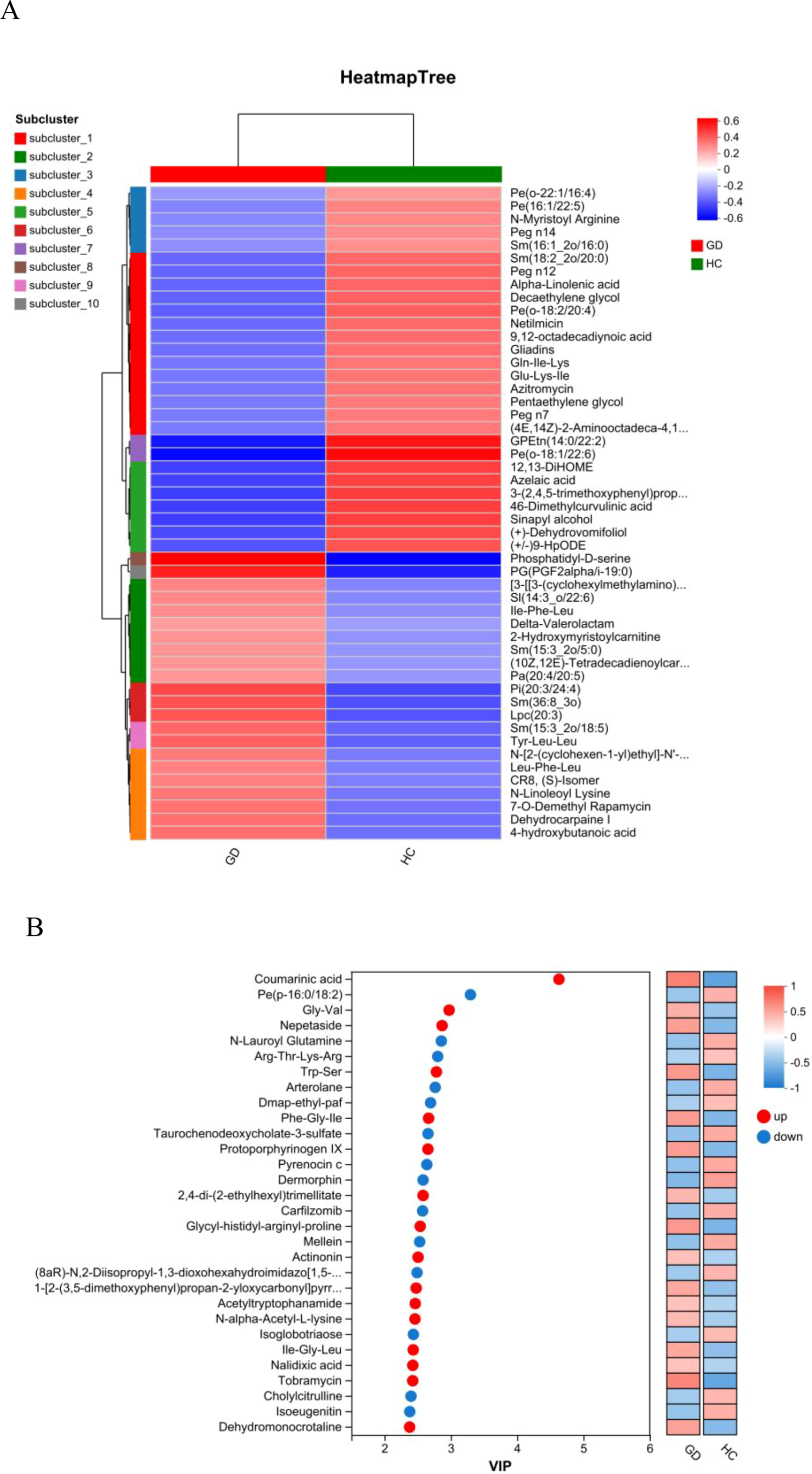
Hierarchical clustering and variable importance analysis. **(A)** Heatmap of hierarchical clustering showing distinct metabolomic patterns between the GD and HC groups, with metabolites grouped into co-expression subclusters. **(B)** VIP plot from the OPLS-DA model ranking the top 30 metabolites contributing to group separation (VIP > 1).

Unsupervised hierarchical clustering was performed to visualize the overall expression patterns of the significantly differential metabolites across all individual samples in the GD and HC groups. The resulting heatmap (**Figure 4A**) clearly segregated the samples into two primary clusters, which corresponded perfectly with the GD and HC groups, thereby providing robust validation of the distinct metabolomic signature of Graves’ disease. Furthermore, the metabolites were clustered into several subclusters (e.g., subcluster_1 to subcluster_10) based on their co-expression patterns. Specific subclusters exhibited coordinated upregulation or downregulation in the GD group. For instance, metabolites such as alpha-linolenic acid, 12,13-DiHOME, azelate acid, and various phospholipids [e.g., PE(16:1/22:5), PE(o-18:1/22:6)] showed distinct expression trends. This coordinated regulation within metabolite subclasses suggests potential functional linkages and common regulatory mechanisms underlying the metabolic perturbations in GD. The heatmap thus offers a comprehensive overview of the systematic metabolic changes and identifies groups of metabolites that may play synergistic roles in the disease’s pathophysiology.

To identify the metabolites that contributed most significantly to the separation between the GD and HC groups observed in the OPLS-DA model, a VIP analysis was conducted. The VIP plot (**Figure 4B**) ranks these influential metabolites based on their VIP scores, with a score greater than 1.0 typically considered significant for group discrimination. The top-ranking metabolites with the highest discriminatory power included coumarinic acid, the phospholipid PE(p-16:0/18:2), the dipeptide Gly-Val, and nepetaside. Other notable high-VIP metabolites encompassed compounds such as N-lauroyl glutamine, the tetrapeptide Arg-Thr-Lys-Arg, acetyltryptophanamide, and taurochenodeoxycholate-3-sulfate. This list of high-VIP metabolites, which includes lipids, amino acid derivatives, and bile acids, provides a prioritized set of the most reliable candidate biomarkers that are most responsible for the metabolic distinction of Graves’ disease, guiding further targeted investigation and potential clinical application.

**Figure 5:**
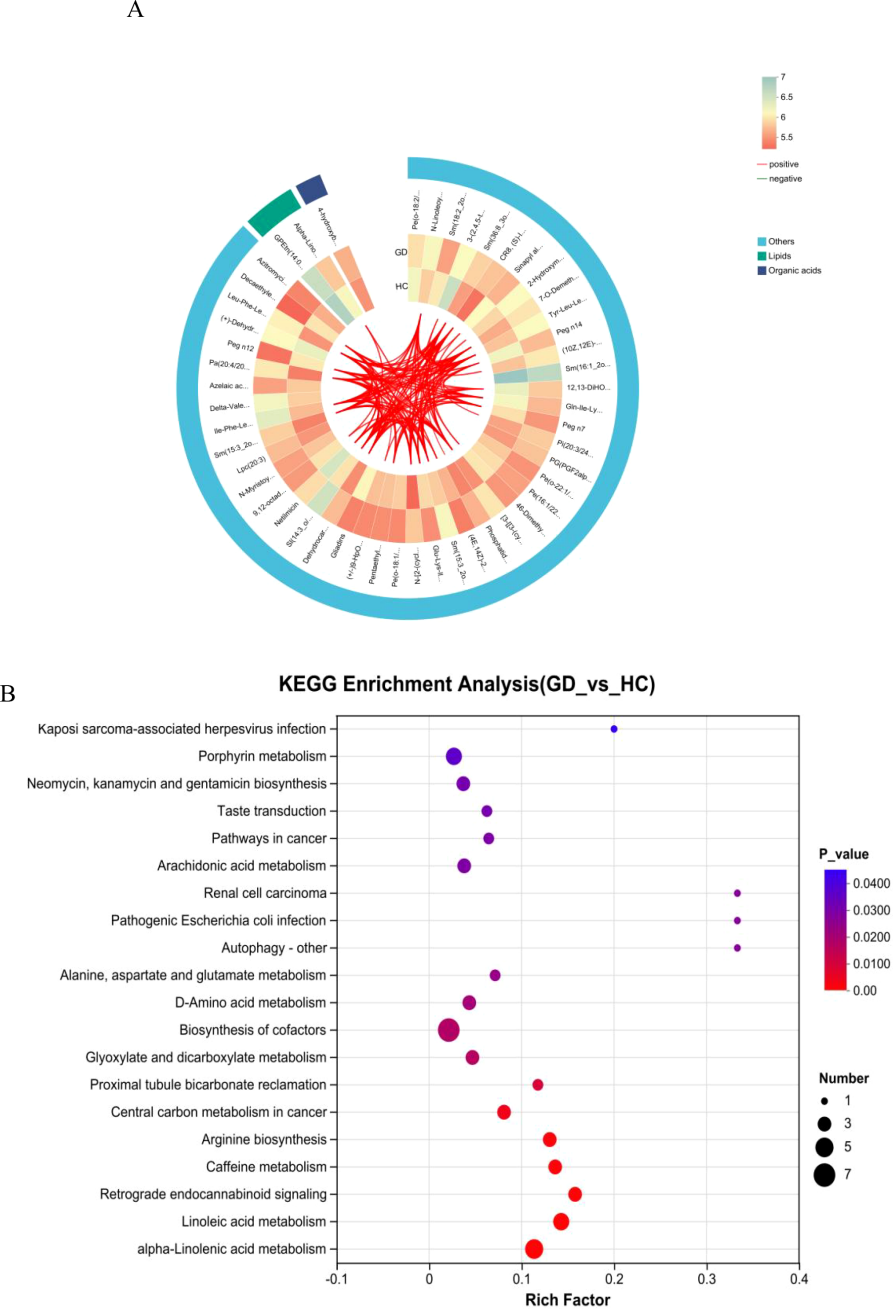
Correlation network and pathway enrichment analysis. **(A)** Chord diagram depicting correlation networks among significantly altered metabolites, grouped into lipids, organic acids, and others. **(B)** Bubble plot of the KEGG pathway enrichment analysis showing the most significantly altered pathways in GD.

A correlation network analysis was performed to visualize the complex interrelationships among the significantly altered metabolites in GD. The resulting chord diagram (**Figure 5A**) illustrates the extensive co-regulation patterns, where metabolites are categorized into major classes, predominantly lipids and organic acids, with the remaining compounds grouped as others. The diagram reveals dense clusters of connections within and between these categories, particularly among lipid species. This intricate network of positive and negative correlations indicates a highly coordinated metabolic response in Graves’ disease. The central role of lipid metabolites in the network, interacting strongly with each other and with organic acids, suggests that dysregulation of lipid metabolism forms a core hub in the pathophysiology of GD. Thi systemic view of metabolic interactions provides insights beyond individual biomarkers, highlighting disrupted functional modules and potential key regulatory nodes in the disease’s metabolic network.

### 3.6 Correlation network and pathway enrichment analyses uncover coordinated metabolic dysregulation

KEGG pathway enrichment analysis was performed to systematically identify biological pathways that were significantly altered in Graves’ disease. The bubble plot (**Figure 5B**) visualizes the results, where the size of the bubbles corresponds to the number of differential metabolites mapped to a pathway, and the color represents the statistical significance (−log10(*p*-value)). The rich factor indicates the proportion of differential metabolites found in a given pathway relative to all metabolites annotated to that pathway.

The analysis revealed significant enrichment in several key metabolic pathways. Most notably, linoleic acid metabolism and alpha-linolenic acid metabolism were among the top enriched pathways, underscoring a major disruption in the metabolism of essential polyunsaturated fatty acids. Arachidonic acid metabolism, a crucial pathway for inflammatory signaling, was also highly enriched, aligning with the autoimmune and inflammatory nature of GD. Pathways in alanine, aspartate, and glutamate metabolism and arginine biosynthesis were significantly altered, highlighting recurrent perturbations in amino acid metabolism. Other enriched pathways included biosynthesis of cofactors and glyoxylate and dicarboxylate metabolism.

Interestingly, several enriched pathways were related to specific human diseases or infections (e.g., Kaposi sarcoma-associated herpesvirus infection, pathogenic *Escherichia coli* infection); these likely represent shared signaling or metabolic modules rather than a direct etiological link. The collective enrichment results strongly suggest that GD is characterized by profound dysregulation in lipid inflammatory pathways and specific amino acid metabolic routes.

**Figure 6:**
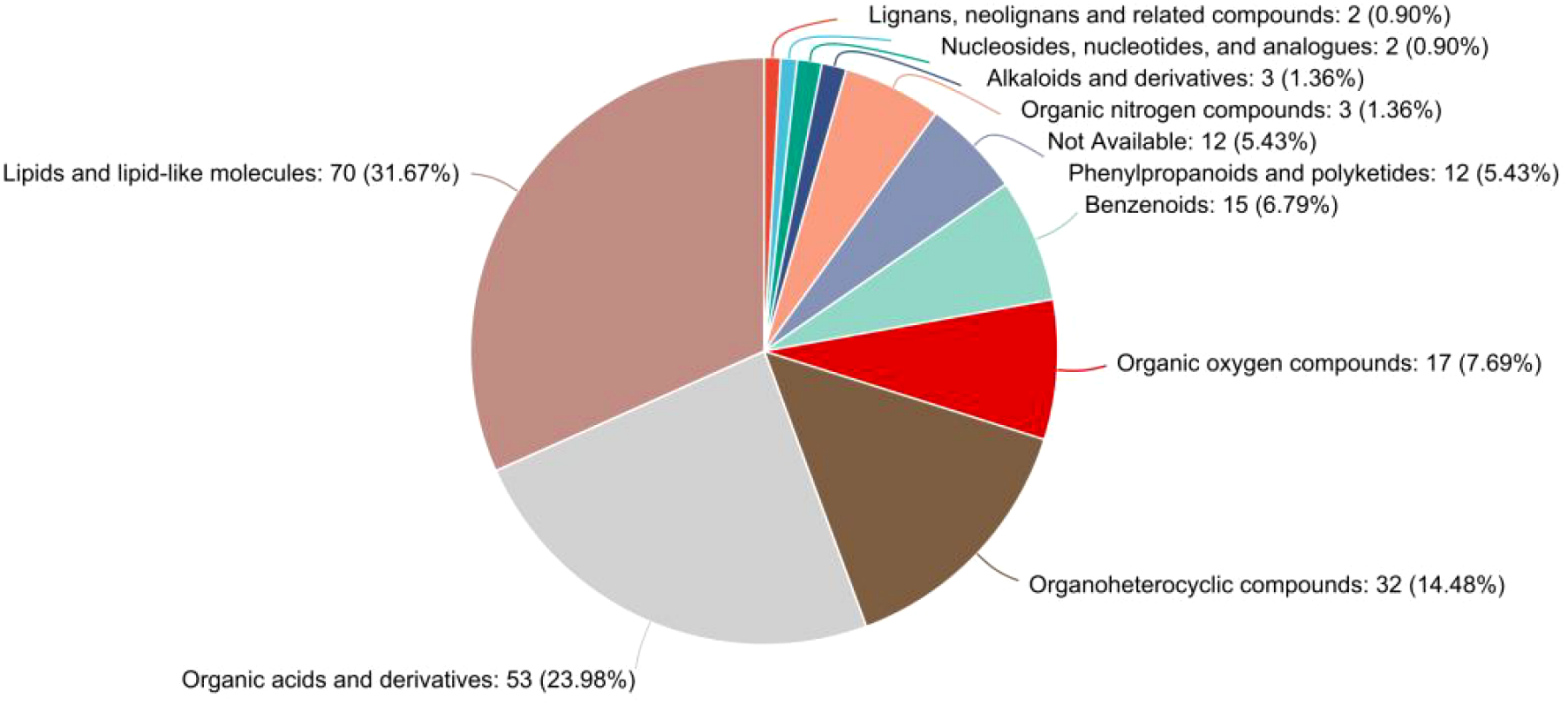
Pie chart detailing the HMDB superclass distribution of metabolites identified as significantly different between the GD and HC groups. The classification is overwhelmingly dominated by “lipids and lipid-like molecules” (31.67%) and “organic acids and derivatives” (23.98%), providing definitive evidence for the central role of these compound classes in the metabolic dysregulation of Graves’ disease. The chart lists the percentage and count of metabolites for each superclass.

The classification of significantly differential metabolites based on the HMDB superclass system was further refined and confirmed, as illustrated in the pie chart (**Figure 6**). This analysis provided a precise quantitative breakdown, unequivocally showing that lipids and lipid-like molecules constituted the largest proportion of altered metabolites, accounting for 31.67% (70 out of the 221 HMDB-annotated differential metabolites) of the total in this annotated subset. The second largest superclass was organic acids and derivatives, representing 23.98% (53 metabolites). Together, these two superclasses dominated the metabolic profile of GD, comprising over 55% of all identified differential metabolites. This finding robustly corroborates the results from prior KEGG and chemical class analyses, solidifying the conclusion that perturbations in lipid and organic acid metabolism are a fundamental characteristic of GD. Other notable superclasses included organoheterocyclic compounds (14.48%), benzenoids (6.79%), and organic oxygen compounds (7.69%). A small fraction of metabolites (5.43%) remained unclassified (not available). This detailed HMDB taxonomy offers a chemically grounded, high-level overview that powerfully emphasizes the specific types of biochemical compounds most affected in GD.

### 3.7 ROC and variable importance analyses validate the diagnostic potential of key metabolites

**Figure 7:**
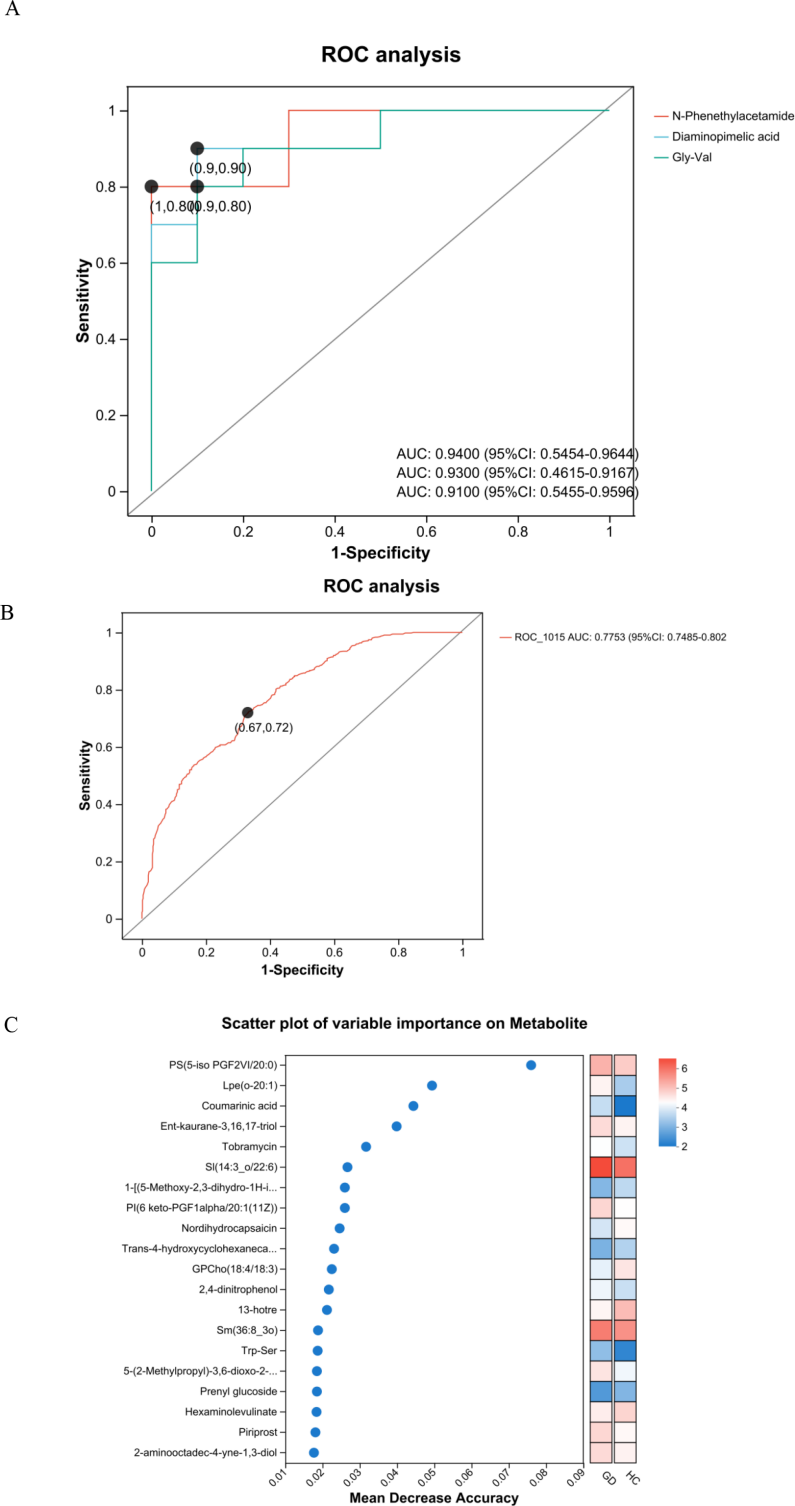
Receiver operating characteristic (ROC) curves and variable importance scatter plot. **(A)** ROC curves of candidate serum metabolites for discriminating patients with Graves’ disease from healthy controls. **(B)** ROC curve for the multimetabolite panel combination, showing an AUC of 0.775 (95% CI: 0.749–0.802). **(C)** Scatter plot of variable importance based on mean decrease accuracy.

To evaluate the diagnostic performance of the identified differential metabolites, ROC curve analysis was performed for both individual candidates and a metabolite panel (**Figure 7**). The ROC curves for several top candidate biomarkers demonstrated strong discriminatory power between GD patients and healthy controls (**Figure 7A**). The metabolite N-phenethylacetamide exhibited the highest individual diagnostic accuracy (AUC = 0.9400; 95% CI: 0.5454–0.9644). Diaminopimelic acid also showed excellent performance, with an AUC of 0.9300 (95% CI: 0.4615–0.9167). Similarly, the dipeptide Gly-Val demonstrated high diagnostic potential with an AUC of 0.9100 (95% CI: 0.5455–0.9596). Furthermore, to explore the potential for enhanced diagnostic performance, a combined model incorporating key metabolites was constructed. This multimetabolite panel yielded an AUC of 0.775 (95% CI: 0.749–0.802) (**Figure 7B**). The sensitivity and specificity values at various thresholds for these biomarkers further support their clinical utility. Collectively, the ROC analysis underscores the validity of our metabolomic approach in identifying biomarkers with significant diagnostic potential for Graves’ disease, both individually and in combination.

Variable importance analysis was performed to rank metabolites based on their contribution to distinguishing Graves’ disease patients from healthy controls (**Figure 7C**). The mean decrease in accuracy was used as the metric to evaluate the discriminatory power of each metabolite. Key metabolites with the highest importance scores included PS(5-iso PGF2VI/20:0), Lpe(o-20:1), coumarinic acid, Ent-kaurane-3,16,17-triol, and SI(14:3_o/22:6). Additional metabolites such as nordihydrocapsaicin, GPCho(18:4/18:3), 2,4-dinitrophenol, Trp-Ser, and piriprost also demonstrated significant importance in the classification model. This analysis highlights specific metabolites that play crucial roles in the metabolic differentiation of GD, providing valuable candidates for further biomarker validation and mechanistic studies.

## 4 Discussion

This LC-MS-based (3) serum metabolomic study provides a comprehensive profile of the profound metabolic disturbances present in patients with untreated Graves’ disease from the Shenzhen region. We identified 334 significantly dysregulated metabolites, with a predominant disruption in lipid and organic acid metabolism pathways. Among the candidate biomarkers validated by ROC analysis, N-phenethylacetamide exhibited the highest individual diagnostic accuracy (AUC = 0.94). Diaminopimelic acid also showed exceptional diagnostic performance (AUC = 0.93), positioning it as another top-tier candidate. The high diagnostic accuracy of diaminopimelic acid warrants further investigation (17). As a key intermediate in the lysine biosynthesis pathway in bacteria and plants, its presence and dysregulation in human serum suggest potential involvement of gut microbiota or novel host metabolic pathways in GD (18). While its specific role in human pathophysiology is less defined, its significant alteration highlights a previously overlooked aspect of GD metabolism that merits deeper exploration (19). Concurrently, the dipeptide Gly-Val demonstrated robust diagnostic performance (AUC = 0.91) and may hold particular mechanistic significance (20).

The most striking finding of our study is the predominant dysregulation of lipid and organic acid metabolism in GD patients. This widespread dysregulation of fundamental metabolic pathways provides a rich source of diagnostic signals, forming the foundation for the high accuracy of our candidate biomarkers. Furthermore, the combination of these metabolites into a panel, while yielding a currently modest AUC of 0.775, underscores the complex, multifactorial nature of GD and points to the potential of a multi-analyte approach for capturing the disease’s heterogeneity, a strategy that may be refined in larger cohorts. Metabolites belonging to the superclasses of “lipids and lipid-like molecules” and “organic acids and derivatives” constituted over 55% of all significantly altered compounds (21). This observation aligns with previous studies that have reported lipid metabolic disturbances in GD (8), but our untargeted approach provides a more comprehensive picture of the specific lipid species affected (22). The significant enrichment of pathways related to linoleic acid, alpha-linolenic acid, and arachidonic acid metabolism is particularly noteworthy (23). These polyunsaturated fatty acids are precursors to various inflammatory mediators, and their dysregulation strongly supports the involvement of enhanced inflammatory signaling in GD pathogenesis. The alteration in arachidonic acid metabolism, especially, provides a direct metabolic link to the autoimmune and inflammatory processes that characterize Graves’ disease (24).

Beyond the metabolic pathways identified in our study, it is important to consider the potential interplay with other systemic regulators of immunity, such as vitamin D and the gut microbiome. Vitamin D is a well-established immunomodulator, and its deficiency has been linked to an increased risk of various autoimmune diseases, including potentially modulating β-cell activation and antibody production (25). Furthermore, the gut microbiome exerts a profound influence on host immunity and metabolism. The microbial synthesis of short-chain fatty acids and other metabolites can shape the immune landscape, and dysbiosis has been implicated in the pathogenesis of autoimmune conditions (18). While our current LC-MS-based serum metabolomics approach did not directly measure vitamin D levels or microbial compositions, the profound dysregulation of host metabolism we observed, particularly in lipids and organic acids, may very well be intertwined with the status of the gut microbiome and vitamin D metabolism. This represents a compelling avenue for future research, integrating metabolomic, microbiomic, and micronutrient analyses to build a more comprehensive model of Graves’ disease pathophysiology.

In addition to lipid metabolism, our pathway analysis revealed significant perturbations in amino acid metabolic pathways, including alanine, aspartate, glutamate metabolism, and arginine biosynthesis (26). These findings corroborate earlier reports of amino acid metabolism alterations in GD (7) and suggest a complex reprogramming of nitrogen metabolism that may support the increased metabolic demands and immune activation associated with hyperthyroidism (27). Our untargeted platform allowed for a more detailed characterization of the specific lipid species involved in GD. While previous studies noted broad changes in phospholipids, we identified distinct plasmalogen species, such as PE(P-16:0/18:2), as being significantly altered (28). Plasmalogens are ether phospholipids with antioxidant properties and roles in signal transduction, and their specific depletion may indicate increased oxidative stress or specific membrane remodeling in GD, offering a new layer of mechanistic insight beyond the well-known inflammatory polyunsaturated fatty acids (PUFAs) (29).

The composition of our cohort, consisting exclusively of treatment-naive patients from a specific geographical region (Shenzhen), may account for certain unique aspects of our metabolic signature (30). Differences in diet, environment, and genetic background compared to cohorts from other regions could influence the metabolome (31). By providing a baseline profile free from the confounding effects of antithyroid medications (32), our data serve as a valuable reference for future studies investigating treatment responses or regional variations in GD. We hypothesize that these metabolic changes are not merely consequences but may actively participate in GD pathogenesis. The dysregulation of Gly-Val could fuel clonal expansion of autoreactive T and B lymphocytes by providing a readily available source of glycine and valine, crucial for protein synthesis and immune cell activation (24, 26). Concurrently, the upregulation of arachidonic acid metabolism likely contributes a pervasive pro-inflammatory milieu through the production of eicosanoids (23), while the broader disruption in amino acid metabolism may simultaneously reflect the hypermetabolic state and supply biosynthetic precursors for immune escalation (27). This creates a potential feed-forward cycle where inflammation and metabolic reprogramming mutually reinforce each other. Although the primary focus of this study is biomarker discovery, these proposed mechanisms offer testable hypotheses for future research into how specific metabolites like Gly-Val might mechanistically contribute to GD progression.

Several limitations of this study should be acknowledged (19). First, the sample size, while sufficient for initial biomarker discovery, requires expansion in future validation studies (33). Second, our cross-sectional design cannot establish whether the observed metabolic changes are causes or consequences of GD (34). Longitudinal studies tracking metabolic changes during treatment and remission would help address this question (35). Additionally, the untargeted metabolomics approach, while powerful for hypothesis generation, carries the inherent risk of false-positive identifications or isobaric interferences, necessitating that the biological relevance of all putative biomarkers be interpreted with caution and confirmed through targeted validation (21, 22). Finally, while we identified numerous significantly altered metabolites, the biological functions of some compounds remain to be fully characterized.

## 5 Conclusion

This study robustly confirms the profound dysregulation of inflammatory lipid and amino acid metabolic pathways in Graves’ disease. Simultaneously, it unveils a panel of novel, high-performance biomarker candidates, with N-phenethylacetamide, diaminopimelic acid, and the dipeptide Gly-Val emerging as the most discriminative. By integrating our findings with the existing literature, we not only reinforce the consistent metabolic core of GD but also delineate the unique contribution of our work: the identification of this specific triad of biomarkers with exceptional diagnostic potential. We acknowledge the limitations inherent in this study, including its sample size and the exploratory nature of the proposed mechanistic hypotheses. Nevertheless, these findings establish a refined metabolic framework for GD and generate compelling, testable hypotheses for future research. The critical next step is to experimentally determine whether these metabolites act as immunomodulatory signals that potentiate T-cell activation, influence β-cell antibody production, or alter thyrocyte susceptibility to immune attack. Employing advanced techniques such as stable-isotope tracing in primary immune cells and genetic manipulation of metabolic enzymes will be pivotal to dissecting the underlying cause–effect relationships. Ultimately, deciphering why the GD metabolome is reconfigured in this specific manner will unlock fundamental biological insights into disease etiology that extend far beyond the initial scope of biomarker discovery.

## Data Availability

The data has been uploaded to the Metabolomics Workbench with DataTrack ID 6263, which can be accessed at: https://www.metabolomicsworkbench.org/data/DRCCDataDeposit.php?Mode=SetupListDataUpload&UploadMode=ListDataUpload.5.
